# The Biogeography of Putative Microbial Antibiotic Production

**DOI:** 10.1371/journal.pone.0130659

**Published:** 2015-06-23

**Authors:** Hélène Morlon, Timothy K. O'Connor, Jessica A. Bryant, Louise K. Charkoudian, Kathryn M. Docherty, Evan Jones, Steven W. Kembel, Jessica L. Green, Brendan J. M. Bohannan

**Affiliations:** 1 Institut de Biologie, UMR CNRS 8197, Ecole Normale Supérieure, Paris, France; 2 Department of Ecology and Evolutionary Biology, University of Arizona, Tucson, Arizona, United States of America; 3 Civil and Environmental Engineering, Massachusetts Institute of Technology, Cambridge, Massachusetts, United States of America; 4 Department of Chemistry, Haverford College, Haverford, Pennsylvania, United States of America; 5 Department of Biological Sciences, Western Michigan University, Kalamazoo, Michigan, United States of America; 6 Institute of Ecology and Evolution, University of Oregon, Eugene, Oregon, United States of America; 7 Département des sciences biologiques, Université du Québec à Montréal, Montréal, Québec, Canada; Wageningen University, NETHERLANDS

## Abstract

Understanding patterns in the distribution and abundance of functional traits across a landscape is of fundamental importance to ecology. Mapping these distributions is particularly challenging for species-rich groups with sparse trait measurement coverage, such as flowering plants, insects, and microorganisms. Here, we use likelihood-based character reconstruction to infer and analyze the spatial distribution of unmeasured traits. We apply this framework to a microbial dataset comprised of 11,732 ketosynthase alpha gene sequences extracted from 144 soil samples from three continents to document the spatial distribution of putative microbial polyketide antibiotic production. Antibiotic production is a key competitive strategy for soil microbial survival and performance. Additionally, novel antibiotic discovery is highly relevant to human health, making natural antibiotic production by soil microorganisms a major target for bioprospecting. Our comparison of trait-based biogeographical patterns to patterns based on taxonomy and phylogeny is relevant to our basic understanding of microbial biogeography as well as the pressing need for new antibiotics.

## Introduction

How and why the functional traits of organisms (characteristics of an organism that are linked with its fitness or performance) vary across communities, space, and environmental gradients remains an important unanswered question [1–6]. A trait-based perspective on biogeography can reveal which processes shape the composition and function of ecological communities, help forecast how communities will respond to environmental change, and guide bioprospecting efforts. While interest in trait-based biogeography has motivated efforts to compile global trait databases, traits are poorly sampled in species-rich and ecologically important groups such as plants, plankton, insects, and microorganisms [4, 6, 7]. The current incompleteness of trait databases is a major limitation, impeding our ability to build global ecosystem, bioclimatic, and biogeochemical models.

In this paper, we apply likelihood-based character reconstruction techniques to the emerging field of trait-based microbial biogeography [6–9]. Microbes play fundamental roles in ecosystem function, in particular through mediating global biogeochemical cycles [10]; yet we know very little about how and why microbial functional diversity varies spatially. This is due in part to difficulty in assessing trait diversity for the majority of microbes that cannot be easily cultivated in the laboratory.

Current cultivation-independent approaches to estimating microbial functional biogeography include analysis of groups thought to play an important role in ecosystem processes, such as ammonia-oxidizing bacteria, amplified with the 16S rRNA taxonomic gene [11], analysis of PCR-amplified target genes mediating specific steps in biogeochemical cycles [12], microarray-based genomic technology such as the GeoChip [8, 9, 13], and genomics-enabled approaches such as metagenomics and metatranscriptomics [14, 15]. These methods can identify broad functional categories, such as nitrogen fixation or photosynthesis strategies, but cannot easily identify trait states within these broad categories. For example, these methods can’t identify whether organisms perform denitrification optimally at different pHs, temperatures or substrate concentrations. Another limitation of commonly used cultivation-independent approaches is that function can often be assigned only to characterized genes.

Using our phylogenetic approach, we investigate the trait-based biogeography of soil microbes. We focus on a class of antibiotic production genes—type II polyketide synthases (PKSs, [16])—that have great relevance to human health, as they are the ultimate source of a number of important antibiotics used to combat pathogenic infections. We analyze the diversity and biogeography of potential antibiotic production at unprecedented spatial scales, ranging from centimeters to thousands of kilometers and spanning three continents. We anticipate that our results will help guide bioprospecting strategies for the discovery of new antibiotics [17].

## A general phylogenetic approach to trait-based biogeography

**Fig 1:**
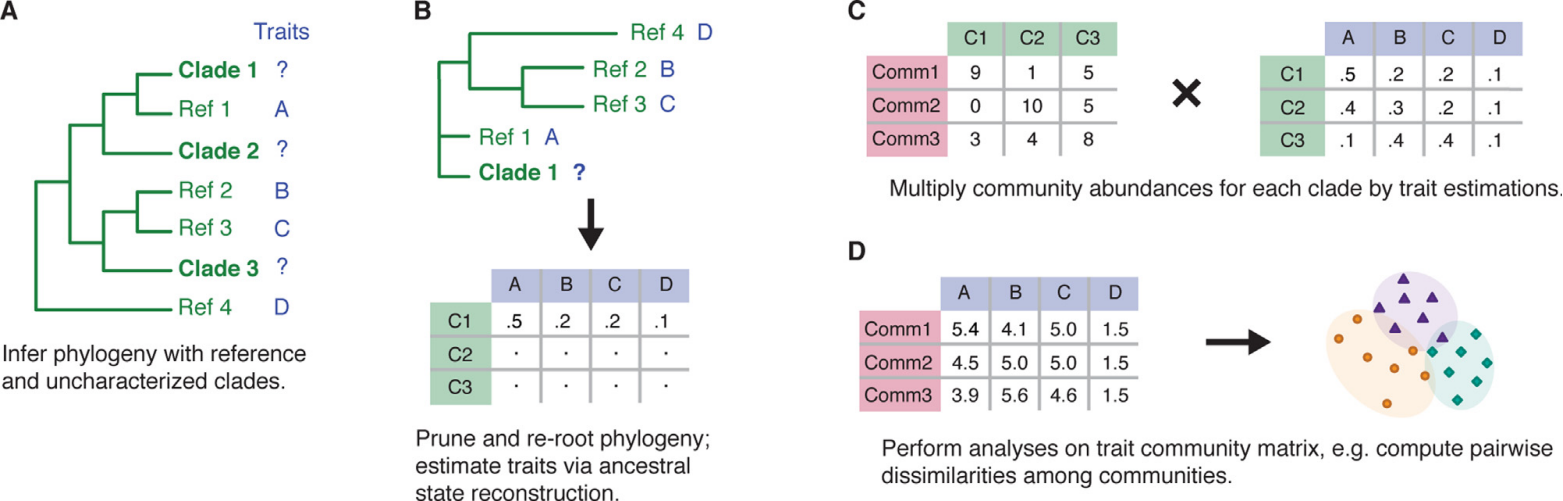
Illustration of the methodological approach used to investigate trait-based biogeography. A) Given a phylogenetic tree with characterized “reference” members (denoted Ref) and uncharacterized members (denoted Clade or C; “clades” can design sequences, individuals or species depending on the study), B) the traits of uncharacterized members can be estimated. C) After rarefying the samples to standardize sampling intensity, the inferred traits can be used to estimate pseudo abundance values for each trait in each community. If the “clades” design sequences or individuals (such as in the present study), the community abundance matrix is in fact a simple presence-absence matrix. D) These pseudo-abundances can then be used for biogeographic analyses. The approach, illustrated here for discrete characters, can readily be adapted to continuous ones. In B, the discrete suite of probability values representing the probability that clade *i* codes for each type would then be replaced by a continuous probability distribution *ϕ*_*i*_(*x*) representing the probability that clade *i* has character *x*. In C, multiplication of the probability distributions corresponding to each clade with the community matrix yields for each community *j* a continuous distribution *ϕ*_*j*_ representing the estimated number of clades with character x. For community 1 for example, this distribution would be given by *ϕ*_1_(*x*) = 9*ϕ*_1_(*x*) + *ϕ*_2_(*x*) + 5*ϕ*_3_(*x*).

One of the biggest limitations in trait-based biogeography for species-rich groups is the paucity of trait databases [4, 6]. Here we present an approach based on phylogenetic character inference methods that can help circumvent this problem (Fig 1). This approach can be applied to any phylogenetic tree describing the relationship among sequences, individuals, or species (thereafter referred to as “clades”). We assume, as is the case in most trait databases, that the trait of interest for some of these clades has been characterized (the “reference” clades), but the trait of others has not (the “uncharacterized” clades, Fig 1A). We infer the traits of the uncharacterized clades using the traits of characterized clades and likelihood-based character reconstruction methods ([18], Material and Methods, Fig 1B). When uncharacterized clades form isolated clusters with no characterized members, no trait can be assigned, indicating that the clade potentially represent new traits. Focusing on such clades can thus guide the discovery of entirely new functional traits.

The uncertainty associated with phylogenetic character reconstruction is often high, particularly when examining microbial phylogenies where reference organisms are scarce. To overcome this difficulty, we adopt a probabilistic approach: instead of assigning a single trait to uncharacterized clades, we describe the trait by a suite of values, each of which represents the probability that the clade has the corresponding trait (Material and Methods, Fig 1B). Next we estimate the abundance of each trait in each community. If the phylogeny represents relationships among higher groupings (e.g. species in the case of macroorganisms or clades of related genes in the case of microorganisms), the estimate is obtained by summing the probabilities associated with the trait across groups from the community, weighted by their abundance (Fig 1C and 1D). Alternatively, if the phylogeny represents relationships among individuals or sequences, the estimate is obtained by summing the probabilities associated with the trait across individuals or sequences from the community, to account for intraspecific variability [5]. The resulting pseudo-abundance community matrix can then be used for community analyses, such as computing trait diversity within and between samples (Fig 1D).

## Measuring the trait diversity of type II PKSs

We applied the general phylogenetic approach above to analyze the potential diversity of type II polyketide antibiotics in soils. Soil microorganisms, in particular Actinomycetes, produce a variety of type II polyketide antibiotics that are thought to play a major role in microbial defense and communication [19]. Such antibiotics can directly affect an organism’s fitness, and thus antibiotic production can be considered a “trait” in the classic sense [2, 7]. Ketosynthase alpha (KSα) is a requisite part of type II PKS gene clusters that encodes suites of enzymes responsible for producing spore pigments and a wide variety of polyketide antibiotics. KSα is commonly used to estimate type II PKS diversity in soil [16, 20–24].

We sampled soil cores in the Mediterranean climate shrublands of Australia, Chile, and South Africa. Fifty samples were taken on each continent, separated by geographic distances ranging from 1 cm to 170 km (Material and Methods). We measured a variety of environmental variables characterizing the environmental conditions in which the soil samples were collected (Material and Methods) and recorded the presence-absence of woody plants in 20 x 20 m quadrats surrounding the soil cores (see [25] for details). In contrast to several recent studies [23, 24], we sampled in similar habitats across continents to increase the power to detect biogeographic patterns due primarily to geographic distance. KSα genes were amplified from our soil samples using PCR, cloned, and sequenced (Material and Methods). This resulted in a total of 11,732 KSα sequences, 573 bp long, evenly distributed across 144 samples.

Following the general phylogenetic approach outlined above, we assembled a database of KSα reference sequences. We found 70 KSα reference sequences with structurally characterized products: 7 are involved in spore pigment synthesis, and 63 are involved in the synthesis of a set of 58 unique antibiotic molecules that can be grouped into 20 different chemotypes (or groups of related chemical structures [19], Material and Methods & S1 Table).

We constructed a phylogenetic tree describing the evolutionary relationships among the environmental sequences and those of the reference database (Fig 1A, Material and Methods, S2A Fig). The environmental sequences span the entire reference database phylogeny, suggesting that the set of primers we used did not miss any major part of the known KSα diversity. Using the phylogenetic tree of environmental and reference sequences, we assigned putative product chemotypes to each environmental sequence (Fig 1B, Material and Methods). We first identified sequences that most likely encode for spore pigments (S2B Fig) and, since our principal interest is in describing the biogeography of polyketide antibiotic production, we excluded these putative spore pigment sequences (235 out of 11,732) from further analyses. We then assigned putative chemotypes to each remaining KSα sequence (S2C Fig).

**Fig 2:**
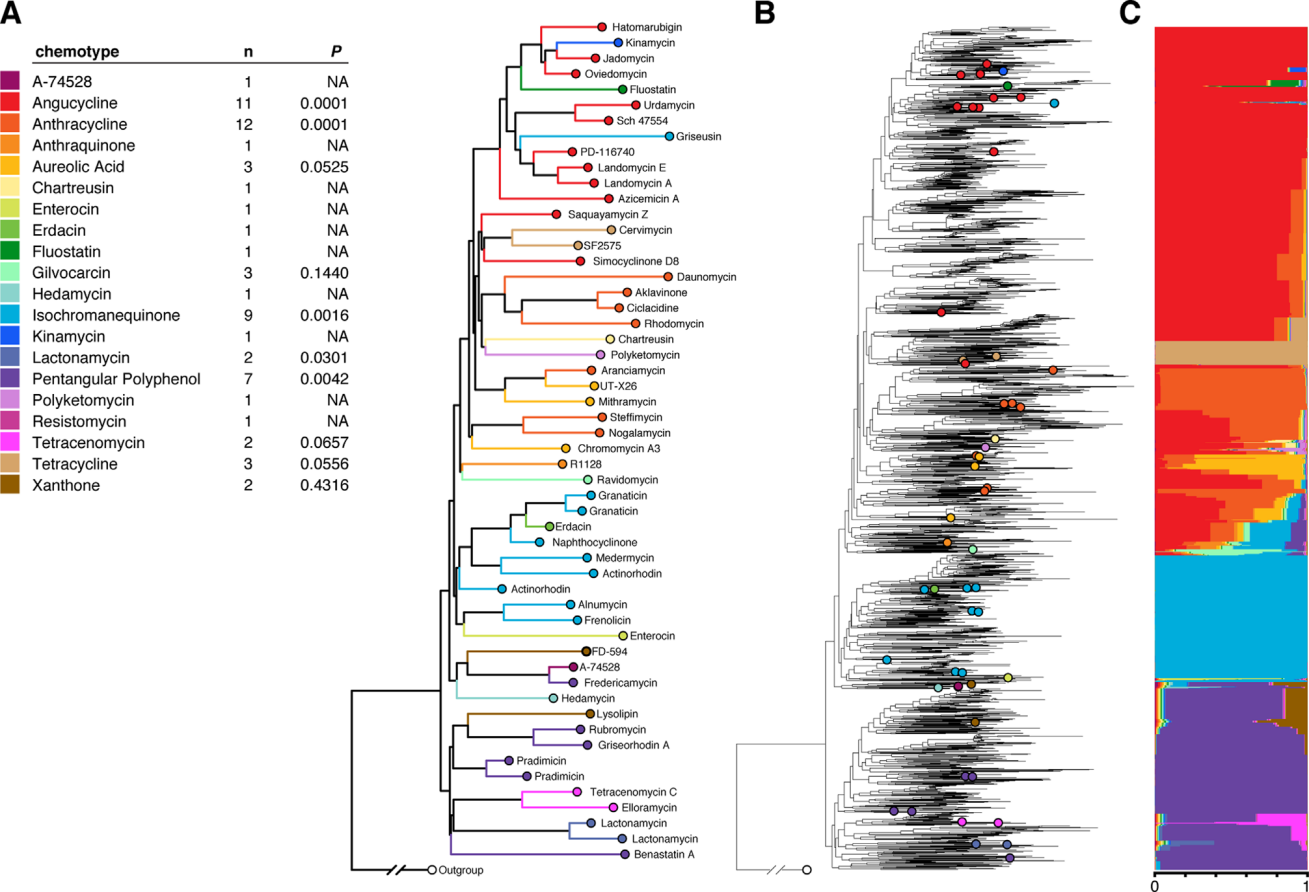
Estimating putative antibiotic production. A) Phylogeny of reference sequences, obtained by pruning environmental sequences from B. Tips are labeled by the polyketide produced and colored by polyketide chemotype. n denotes the number of reference sequences of the given chemotype, and *P* reflects the clustering of the chemotype on the phylogeny (computed as a z-score, see Material and Methods). The clustering is significant or marginally significant for almost all chemotypes for which it could be computed. B) The phylogeny of environmental KSα genes (black), along with reference sequences (colored), allows estimating for each environmental sequence and each chemotype the probability that the sequence codes for the chemotype. C) Each colored stripe indicates the inferred probability that the corresponding sequence codes for the chemotype represented by the color.

The majority of sequences (7,317 out of 11,497) formed a well-supported clade with KSα genes from antibiotic-producing PKS clusters (S2A Fig, top part of the tree, reproduced in Fig 2). In this part of the tree, most sequences (80%) could be assigned to a given chemotype with ≥ 0.75 confidence. The assignment was robust to uncertainties in phylogenetic construction (Material and Methods, S3 Fig). The top third of the phylogeny did not include many reference sequences, which may have led to the high probabilities of the angucycline trait. Some clades in this region of the tree are substantially divergent from reference members and therefore may produce bioactive molecules with novel molecular structures instead of angucycline.

Another group of sequences formed a large, globally distributed clade (comprised of more than 4,000 sequences) that had no antibiotic-producing reference members (S2A Fig, bottom part of the phylogeny). Sequences in this clade had BLAST matches within both cultured and uncultured bacteria, but not outside of bacteria. Hits to cultured bacteria were typically Actinomycetes, and most often *Streptomyces*. Sequences in this clade likely belong to PKS gene clusters for which the products have yet to be characterized. We conducted biogeographic analyses both with and without this large uncharacterized clade.

## Biogeography of type II PKSs based on sequence groups, phylogeny, and traits

We focused our analysis on three aspects of PKS community composition: sequence similarity groups, phylogeny, and traits. The composition in terms of sequence similarity groups (thereafter referred to as “sequence groups”) refers to groups defined on the basis of KSα sequence similarity, as described by Reddy *et al*. [22], and does not account for the phylogenetic relationships among these groups. This composition is summarized by the community matrix describing the presence/absence of sequences across communities with the matrix describing the assignment of each environmental sequence to a specific sequence group. Phylogenetic composition accounts for the phylogenetic relationships among the sequence groups [26]. This composition is summarized by the community matrix and the phylogeny describing the evolutionary relationships among sequence groups. Trait composition refers to the putative chemotypes present across communities; this composition is summarized by the “trait community matrix” obtained by multiplying the matrix describing the presence/absence of sequences across communities with the matrix describing the chemotype estimation for each environmental sequence, derived from the assignment technique described above (Fig 1C and 1D). Different sequence groups can encode the same chemotype, such that the diversity of chemotypes is typically lower than the diversity of sequence groups.

Our probabilistic, phylogenetically-informed approach to estimating traits differs from other methods of inferring polyketide production from KSα sequences. eSNaPD [27] and NaPDoS [28] use sequence homology (either BLAST e-values or % identity) to predict the polyketide products of input sequence, without an indication of assignment confidence. The idea of placing environmental KSα sequences on a phylogeny of reference sequences with known chemotype has been used before in order to identify sequences potentially encoding new chemotypes [29, 30], but it had not yet been developed into a systematic, quantitative estimation of the chemotype produced by environmental sequences.

We characterized beta-diversity, i.e. the turnover in community composition across our samples, for these three dimensions of diversity. We used abundance-weighted dissimilarity metrics (Material and Methods) to measure differences in the sequence groups, chemotypes, and phylogenetic composition (i.e. an overall measure of the phylogenetic uniqueness of communities) identified at each sampling site.

Dissimilarity values were higher for sequence groups (0.94 +- 0.06, mean and s.d. across all pairwise comparisons) than phylogenetic diversity (0.63 +- 0.08) and higher for phylogenetic than trait diversity (0.34 +- 0.15, S4 and S5 Figs). This ranking of dissimilarity values was consistent across continents and spatial scales (S4 Fig). Microbial communities in Australia and South Africa were slightly more similar to one another than communities in Australia and Chile or South Africa and Chile (S5 Fig), mirroring patterns observed in the aboveground plant communities [25]. Rarefaction analyses showed that phylogenetic and trait dissimilarity values changed little with increasing sequencing effort (i.e. sampling effort) while dissimilarity values in sequence groups decreased slightly (S6 Fig). The shape of the rarefaction curves suggests that the ranking of dissimilarity values across diversity measures would remain robust with increasing sequencing depth. Thus, communities tend to be more similar in terms of phylogeny than in terms of sequence groups, reflecting the fact that sequence groups share a common evolutionary history, and more similar in terms of traits than phylogeny, reflecting the fact that different evolutionary paths can lead to similar chemotypes.

**Fig 3:**
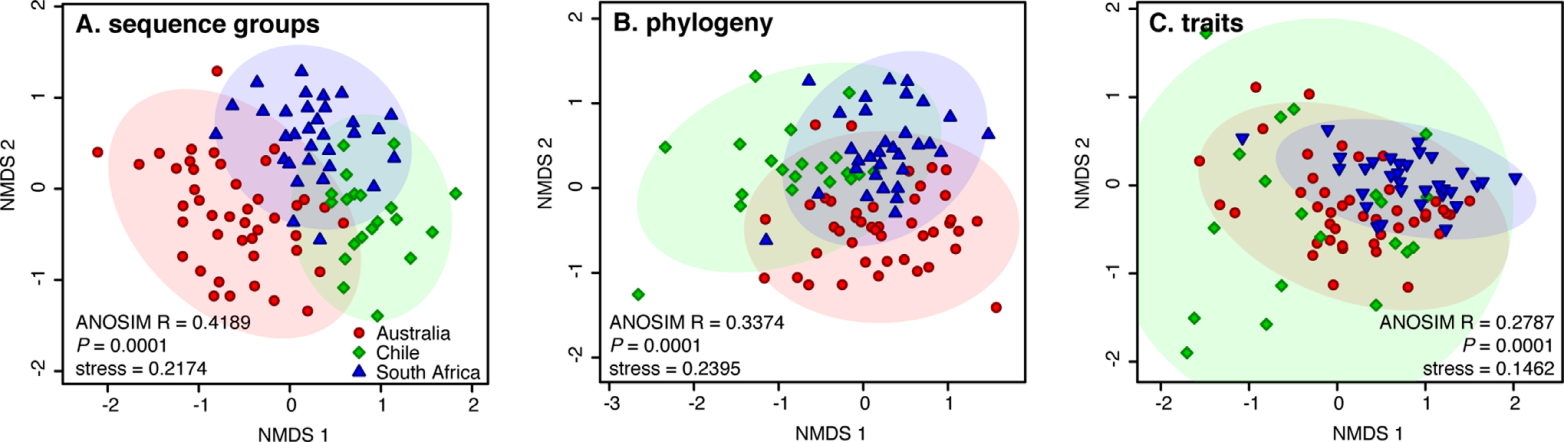
Non-metric multidimensional scaling (NMDS) ordinations based on abundance-weighted dissimilarity metrics reveal that samples cluster by continents. The strength of the clustering is the highest for composition in terms of sequence groups (A), intermediate for phylogenetic composition (B), and the lowest for trait composition (C). Significance values refer to analysis of similarity (ANOSIM) test for differences in community composition among continents. Analyses excluding the large uncharacterized clade (see also S7 Fig).

We observed a significant clustering of communities by continent (Fig 3 and S7 Fig, p<0.0001 in each continent). Communities clustered most strongly when examined with sequence groups; the strength of clustering decreased when community composition was measured with phylogenetic and trait-based diversity metrics (Fig 3 and S7 Fig). This trend is conservative with regards to sequencing depth, given that undersampling is expected to weaken patterns of sequence groups more than phylogenetic and trait-based ones (with equal sequencing effort, traits and phylogenies are more thoroughly sampled than sequence groups). We observed more spatial structure in phylogenetic diversity relative to trait diversity, and more spatial structure for diversity of sequence groups relative to phylogenetic diversity. Uncertainty in trait assignment leads to a homogenization of trait-based beta-diversity values across sites, which may at least in part explain the weak trait-based patterns we observed. Despite this noise, the clustering remained significant for all three measures of biodiversity, demonstrating spatial structure at the global scale.

To quantify the effect of geographic separation on the turnover in community composition, we analyzed the increase in community dissimilarity with geographic distance (Material and Methods, Table 1, S3 and S8 Figs). The increase was most pronounced at the global scale (i.e. when considering cross-continents comparisons) than within-continents, suggesting that the effect of geographic separation is the greatest at large spatial scales. This result should be robust to sequencing depth: undersampling may weaken but not bias spatial patterns. Recent studies suggest that increasing sequencing depth is not crucial to beta-diversity studies [31]. The slope of the increase was generally weak, likely reflecting that we sampled in a relatively homogeneous flora and environment within each Mediterranean-type ecosystem. Despite weak geographic structure, communities were far from demonstrating phylogenetic and trait homogeneity: at least one fourth of the diversity encompassed by any two samples was unique to one of them.

**Table 1:** Drivers of bacterial geographic structure.

	Australia			Chile			South-Africa			Global		
	taxo	phylo	trait	taxo	phylo	trait	taxo	phylo	trait	taxo	phylo	trait
**geography**	0.16	0.17	0.022	0.025	0.028	0.12	**0.24** *	0.21	0.19	**0.39** *	**0.31** *	**0.23** *
**geography alone**	-	-	-	-	-	-	-	-	-	-	-	-
**environment alone**	***	**	*	-	-	-	-	-	-	*	-	-
**vegetation alone**	***	***	**	-	-	-	***	**	-	***	*	-

In the row entitled “geography” numbers represent the slope of the relationship between log_10_-transformed community dissimilarity and log_10_-transformed geographic distance, and stars their significance, computed using Mantel tests (999 permutations per test). The three other rows report the significance of partial regression coefficients from the multiple regressions on distance matrices analysis.

*, p<0.05;

**, p<0.01;

***, p<0.001.

Previous analyses have demonstrated a correlation between KSα diversity and geographic distance or environmental variables, but the relative importance of these factors has not been explicitly tested. We investigated the relative importance of geographic distance, the environment, and plant community composition on bacterial spatial turnover (Material and Methods, Table 1). When accounting for plant and environmental dissimilarity, the effect of geography on bacterial community dissimilarity was no longer significant, even at the global scale. Plant community composition was the strongest predictor of bacterial community composition. Plant community composition was a significant predictor at the global scale and in all continents (except for trait diversity in South Africa and all diversity measures in Chile, where none of the predictors were significant). The combined effect of the environment was the second strongest predictor, but—after accounting for plant dissimilarity and geographic distance—it remained significant only in Australia (all diversity measures) and at the global scale (for diversity in terms of sequence groups). When evaluating the separate effect of individual environmental variables (Material and Methods), plant composition was the only variable standing out as a consistent significant predictor of bacterial community structure.

It is possible that plant community composition has a direct effect on bacterial communities. However, since we collected presence-absence plant data in the distant (20 x 20 m) rather than local surroundings of each bacterial community, it is more likely that the marked correlation between plant and bacterial communities reflects the joint effect of a third geographically-structured component on these communities. We cannot exclude the possibility that this third component corresponds to environmental variables we did not measure, a limitation inherent to beta-diversity studies [32]. However, we measured the main environmental variables thought to have a significant influence on bacterial community composition. Hence, our results suggest that bacterial communities respond to long term, time-integrated environmental conditions (e.g. climate) that may be better reflected by surrounding plant community composition than short-term measures of soil characteristics [33].

## Implications for bioprospecting and trait-based biogeography

Our results suggest that it is more effective to focus bioprospecting efforts on sampling broadly (e.g. across continents) rather than intensively in homogeneous environments spanning only few hundred square kilometers. We identified a large and globally distributed clade distantly related to sequences from known biosynthetic clusters, and smaller clades with only few characterized members. While these clades add uncertainty to our trait-based biogeographic analysis, they are one of our most exciting results: we identified what could be a natural source of novel antibiotics and should be an attractive target for bioprospecting. Heterologous expression of gene clusters with KSα sequences from the least characterized parts of the phylogeny could reveal new antibiotics [34, 35].

The phylogenetic approach for estimating unmeasured traits presented here should support future microbial trait-based biogeography studies. Phylogenetic character reconstruction techniques have been developed to identify microbial populations and their habitat preferences [36], and to analyze the structure and function of ancestral proteins [37]. Our use of character reconstruction differs from these approaches as we use character reconstruction techniques to infer extant rather than ancestral trait states. The authors of PICRUSt [38] also infer extant trait states, although they use an average of the known extant and inferred ancestral traits (weighted by phylogenetic distance) rather than the model-based expectation we use here [18]; in addition, the method is tailored specifically to estimate gene family abundance rather than traits.

Our approach allows for working with a phylogenetic tree rather than genes, contrary to sequence homology techniques. This is particularly useful when phylogenies are built from a series of phylogenetically rather than functionally informative genes, which is often the case in studies of both macro- and microorganisms. Whereas sequence homology would infer traits based on the reference sequence most closely related to the query, our approach considers the integrity of the phylogenetic neighborhood of the query sequence into account and quantifies assignment uncertainty. This vastly improves accuracy, particularly when there are several equally closely related reference sequences with fairly different traits.

A similar approach could be used to assess the biogeography of other important discrete or continuous microbial traits [39]. For example, the trait of interest could be the potential rate of enzyme activity of a particular functional gene variant, although a fair amount of microbial and genetic work is still needed to establish a reference database for enzymatic rates. Genetic variants of *mcr*A, *amo*A, *nir*S or *nif*H reflect different efficiencies of methanogenesis, nitrification, denitrification and N-fixation, respectively. Microbial rhodopsin genes would also lend themselves well to these types of analyses. These genes are distributed across a variety of aquatic and terrestrial environments, and code a suite of different traits important for cell survival, including proton transport (energy generation), chloride transport (maintenance of osmotic balance) and light sensing (phototaxis or gene expression). The approach could also be used to estimate a variety of prototypical microbial functional traits, such as ribosomal copy number [39], GC content, or genome size (see Table 1 from [7] for other examples).

Our phylogenetic approach also has many potential applications in trait-based biogeography in general. Indeed, major trait databases for rich groups such as flowering plants [4] and marine plankton [6] are very sparsely sampled. Approaches to filling in the gaps in these databases are sorely needed [40]. Our approach, based on phylogeny, provides a new angle to the question [41]. Future work aimed at comparing and potentially integrating these different approaches will be highly valuable. Such methods will help infer missing values in trait databases for both macro and microorganisms and map the diversity of traits at large spatial scales. In comparison with traditional biogeographic patterns based on sequence similarity groups, trait-based patterns may be less sensitive, as observed here. However, they also inform much more directly about potential function and responses to environmental change.

## Conclusion

Analyzing the distribution of traits across communities is crucial to understanding both community assembly processes and ecosystem functioning. A solid understanding of microbial trait-based biogeography is particularly relevant for microorganisms, given the key role they play in antibiotic production and biogeochemical cycling. Using phylogenetic information and reference sequences of characterized polyketide synthases, we predicted the putative chemotype of environmental sequences and analyzed the resulting trait-based biogeographic patterns. This allowed us to compare microbial biogeographic patterns based on sequence groups, phylogeny and traits at the global scale. Similar approaches, applied to a variety of taxonomic groups and traits, will ultimately improve our understanding of the spatial distribution of traits, and of ecosystems function, services, and potential response to environmental change.

## Material and Methods

### Soil collection and environmental analyses

We sampled soil in the Mediterranean climate shrublands of Australia, Chile, and South Africa. GPS coordinates are provided in S1 Material & Methods. No specific permissions were required to sample in these locations and sampling did not involve endangered or protected species. These sites were chosen to reduce environmental heterogeneity while sampling at the global scale. On each continent, we collected 50 15-cm deep, 1-cm wide soil cores (150 soil cores total), separated by geographic distances ranging from 1 cm to 170 km (S1 Material & Methods). We also collected a larger amount of soil adjacent to each soil core for analysis of soil texture (% sand, silt and clay). Each soil core was homogenized and stored at -80°C. We measured a total of 15 environmental variables using subsamples of the homogenized soil cores. These variables included moisture, pH, and the concentration of micronutrients (Mg, Al, P, K, Ca, Mn, Fe, Cu, and Zn), and macronutrients (e.g. total N, total C and NO_3_), measured using standard protocols.

### DNA Extraction, PCR, and Sequencing

We extracted DNA from 4 replicates of 200 mg wet mass of each soil core, using a modified version of the protocol by Zhou *et al*. ([42], S1 Material & Methods). We then combined equal volumes of the 4 DNA extracts together for PCR amplification. We performed amplification of the type II PKS KSα gene using primers developed by Metsä-Ketelä *et al*. [16]. We performed 3 replicate PCRs on the pooled DNA, using 1 μL template pooled-DNA per reaction. Details of PCR conditions, DNA clean-up, cloning and sequencing are presented in S1 Material & Methods. Primer and PCR conditions were consistent across all samples, such that potential primer bias shouldn't influence our results.

### Reference sequences, sequence alignment, and tree construction

Reference sequences were found by searching the nucleotide database of GenBank for many variants of the phrase "polyketide synthase" or "ketosynthase alpha", then manually inspecting results for KSα genes, and finally using a BLAST search to find related sequences. Only sequences that had been experimentally implicated in the production of structurally characterized polyketides were included in subsequent analyses, resulting in a total of 79 reference sequences (S1 Table).

We used Clustal through the program BioEdit to align the 11,732 environmental KSα sequences, the 79 structurally characterized reference sequences, and a fabH outgroup ([43], S1 Material & Methods). We then used FastTree 2.1.0 [44] to build a phylogeny of these sequences (GTR branch swapping as suggested by jModelTest 0.1.1 [41]; all other parameters default). This resulted in the construction of a phylogenetic tree with characterized “reference” and uncharacterized “environmental” members (Fig 1A).

### Estimating polyketide products of environmental sequences

The core set of enzymes in a type II polyketide synthase includes a ketosynthase (KSα), a chain length factor (CLF or KSβ), an acyl carrier protein (ACP), and a malonyl-CoA:ACP transacylase (MAT), all of which contribute to the final structure of the polyketide product. Therefore, KSα diversity does not correlate one-to-one with polyketide diversity; nevertheless, closely related KSα sequences tend to contribute to the production of structurally, and potentially functionally similar polyketides ([22], see also our Fig 2). By leveraging this trait conservatism and a maximum likelihood-based trait reconstruction framework, we estimated the characteristics of polyketides produced by our environmental sequences (Fig 1B). We first classified the reference sequences into those coding for spore pigmentation and those coding for antibiotic production. Then, we classified the polyketide products of the reference sequences coding for antibiotic production into chemotypes based upon structural characteristics, chain size, primer unit, and cyclization patterns (S1 Table).

We then estimated the traits of environmental sequences following the method of Garland & Ives [18]: to estimate the traits of an environmental sequence (*e*_*i*_), the global phylogeny was pruned to include only *e*_*i*_ and annotated references. We first tested the clustering of chemotypes on the phylogeny, using a randomization test. For each chemotype, we compared the total phylogenetic diversity (Faith’s PD [45]) spanned by the sequences coding for this chemotype to the PD of 10,000 random draws of the same number of sequences. The significance of the clustering was assessed by computing the ‘z-score’, defined as the rank of the observed PD within random draws, divided by the number of draws + 1. These ‘z-scores’, reported in Fig 2A for each chemotype, indicate that closely related sequences tend to encode the same chemotype, suggesting that phylogeny is informative to predict chemotype (see also [20]). The phylogeny was rerooted at the ancestor of *e*_*i*_ and its closest relative in the reference phylogeny. After rerooting, the states at *e*_*i*_ was estimated using the discrete ancestral character state reconstruction method of Pagel [46] implemented in ape. This likelihood-based method relies on a continuous-time Markov model in which characters evolve with fixed transition rates. We used the “equal-rates” model in which the probabilities to transition from any state to any other state are all equal. For any given environmental sequence, the method provides a suite of probability values, each of which is the probability that the sequence codes for each type.

We tested the robustness of the trait assignment procedure to phylogenetic uncertainty by performing sensitivity analyses on a Bayesian posterior distribution of trees (S1 Material & Methods). Since the trait assignment of a given environmental sequence is computed from a tree containing only that environmental sequence (and all reference sequences), the uncertainty in trait assignment will not increase as more environmental sequences (e.g. arising from next generation sequencing) are included. Rather, the uncertainty will decrease as more reference sequences are included.

All phylogeny manipulations and statistical analyses were performed in the R statistical computing environment version 2.14.1 (R Development Core Team) using functions from the packages picante [47] version 1.2, vegan [48] version 2.0–2, and ape [49] version 2.8. The ancestral state imputation method is implemented in the function phyEstimateDisc, available in version 1.6 of the picante R package [47].

### Defining sequence similarity groups

We defined sequence similarity groups based on the previously built phylogenetic tree of KSα sequences. Specifically, given a fixed % sequence identity cut-off value (x), we traversed the tree from the tips to the root and collapsed all nodes for which all descendant sequences were at least x% similar (R codes are available from the authors). This criterion corresponds to the criterion used in the classical furthest neighbor-clustering algorithm (used, e.g., in mothur, [50]). Although we used the phylogeny to define sequence similarity groups, our measure of diversity in terms of sequence groups does not account for the phylogenetic distance among these groups, as in classical diversity measures based on sequence similarity groups.

Sequences were binned into groups at multiple sequence identity cutoffs to identify the level at which a group included only sequences likely to be involved in production of related polyketides of a single chemotype. Note that the goal here is not to estimate the polyketide products of environmental sequences (which is more accurately done using the ancestral reconstruction approach described above), but rather to quantify the diversity of sequence groups. Below a 90% binning threshold, sequence groups included reference sequences belonging to multiple chemotypes; we thus selected this 90% identity as our threshold to construct groups. Binning the sequences at the 90% level resulted in 2,970 groups. Similarity values for reference KSα sequences associated with the same polyketide product ranged between 99.7 and 100% within sequence groups and between 79.9 and 92.5% among distinct sequence groups.

### Statistical analyses

Communities were rarefied to the minimum number of sequences across samples; the samples that had too few sequences had to be discarded. In the analyses excluding sequences assigned to spore pigment production, this resulted in a rarefaction to 67 sequences per sample across 142 samples (48 samples from Australia, 44 from Chile, and 50 from South-Africa). In the analyses also excluding sequences from the uncharacterized clade, this resulted in a rarefaction to 45 sequences per sample across 95 samples (43 samples from Australia, 20 from Chile, and 32 from South Africa). Analyses were also performed at lower rarefaction levels with more sites included and the results were qualitatively similar. Beta-diversity in terms of sequence groups was measured with Bray-Curtis dissimilarity index, calculated in vegan. Phylogenetic beta-diversity was quantified with the weighted UniFrac metric, computed in PyCogent v 2.6.1 [51]. Trait beta-diversity was computed as the Bray-Curtis dissimilarity index, using the pseudo-abundance matrices resulting from the trait assignment procedure (Fig 1C and 1D). We did not account for variation in PKS copy number variation among bacteria, since our focus was the diversity of KSα gene diversity rather than taxonomic diversity (S1 Material & Methods).

The clustering of samples by continents was analyzed using non-metric multidimensional scaling ordination (NMDS) completed with an analysis of similarities (ANOSIM). The relationship between community similarity and geographic distance was analyzed using Mantel tests. p-values were obtained using 999 permutations in each test and the 95% confidence intervals for the slope of the relationship were computed following Manly [52].

We investigated the relationship between bacterial community similarity, plant community similarity, geographic distance, and environmental distance. Bacterial community similarity and geographic distances were log-transformed before analyses to achieve normality. We scaled environmental variables from 0 to 1, checked them for normality, and transformed them when necessary. Based on the varclust procedure, we dropped the % sand and % silt variables, which were highly correlated with % clay values (ρ^2^ = 0.7). We then used the bioenv procedure to select relevant variables. We computed environmental distances between samples with these variables, using the Euclidian distance. We computed a composite distance of selected environmental variables, and also individual distances corresponding to each variable. To analyze the determinants of bacterial community composition, we used partial Mantel tests [52] and multiple regressions on distance matrices [53]. The procedure was run with the composite environmental measure and each individual environmental variable, for each continent and the global dataset. We used the Bonferroni correction to account for multiple testing. All statistical analyses were performed with the ecodist, Hmisc and vegan R packages.

## Supporting Information

S1 Fig Overview of location and spread of sampling sites. (PDF)

S2 Fig Estimating putative antibiotic production. A) Phylogeny of all environmental KSα genes (black lines), along with antibiotic producing reference sequences (colored dots) and spore pigment producing reference sequences (black dots). B) Each bar indicates the inferred probabilities that the corresponding sequence codes for antibiotic production (in grey) or spore pigment production (in black). For example, a completely grey bar represents a sequence which probability to code for antibiotic production is 1. C) Each bar indicates the inferred probabilities that the corresponding sequence codes for each chemotype represented by the color (i.e. the length of a given colored band is proportional to the probability that the sequences codes for the chemotype represented by the color). Black bars indicate sequences which probability to encode spore pigmentation is greater than 0.5. Sequences from the top part of the tree encode diverse chemotypes. Most sequences from the bottom part of the tree likely encode yet-to-be discovered antibiotics rather than the inferred trait, given the lack of reference sequences in this part of the tree.
(TIF)

S3 Fig Robustness to phylogenetic uncertainty. The trait assignment procedure is robust to phylogenetic uncertainty. A) Distribution across environmental sequences of the proportion of posterior distribution trees assigning the sequence to the most commonly assigned chemotype. For most sequences the most probable chemotype is consistent across phylogenies. B) Boxplot across posterior distribution trees of the number of sequences most probably coding for the given chemotype. “none” indicates that no chemotype was inferred with more than 0.5 confidence. The partitioning of sequences among chemotypes is consistent across phylogenies.
(TIF)

S4 Fig Increase in community dissimilarity with geographic distance within each continent. Comparisons of within-continents increase in community dissimilarity with geographic distance when diversity is measured in terms of sequence groups, phylogeny and traits. Analyses with the large uncharacterized clade excluded (see also S8 Fig)
(TIF)

S5 Fig Community dissimilarity across continents. Boxplots of pairwise dissimilarity values, for measures of diversity based on sequence groups (top panel), phylogeny (middle panel) and traits (bottom panel). A-C: Australia / Chile; A-S: Australia / South Africa; C-S: Chile / South Africa.
(TIF)

S6 Fig Effect of sampling depth on dissimilarity values. Plotted are mean (and 95% CI based on 50 replicate rarefactions) dissimilarity values (across all samples with at least 60 sequences) for taxonomic, phylogenetic and trait-based dimensions of biodiversity, as a function of the rarefaction level. CIs are very tight and can hardly be seen. Dissimilarity values asymptote very quickly with an increasing number of sequences.
(TIF)

S7 Fig NMDS for the full dataset. Results are consistent with results excluding sequences from the uncharacterized clade.
(TIF)

S8 Fig Increase in community dissimilarity with geographic distance within each continent for the full dataset. Results are consistent with results excluding sequences from the uncharacterized clade.
(TIF)

S1 Material & Methods (DOC)

S1 Table Accession number, antibiotic name, chemotype, chain length, first cyclization and priming unit of the reference sequences used in the study. (PDF)
