# Supplementary figures and images for: The Biogeography of Putative Microbial Antibiotic Production
Source: PLoS One. 2015 Jun 23;10(6):e0130659. doi: 10.1371/journal.pone.0130659 (PMC4478008; doi:10.1371/journal.pone.0130659)

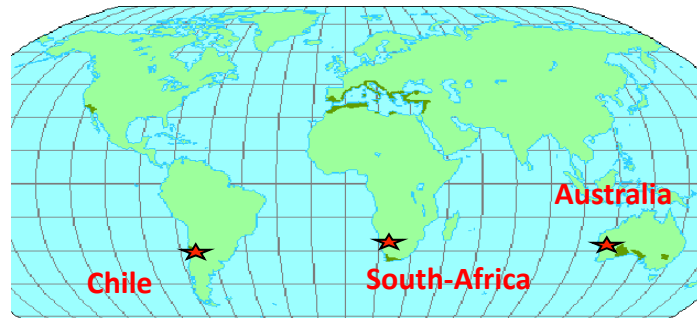

**Chile**

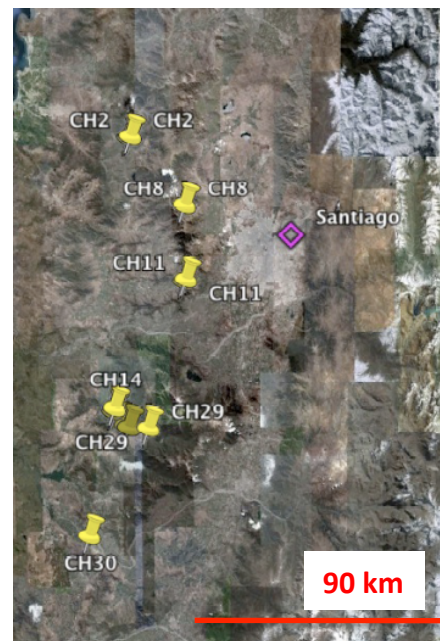

**South-Africa**

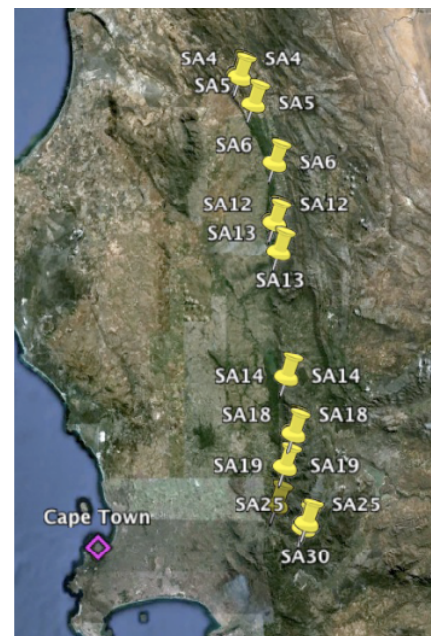

**Australia**

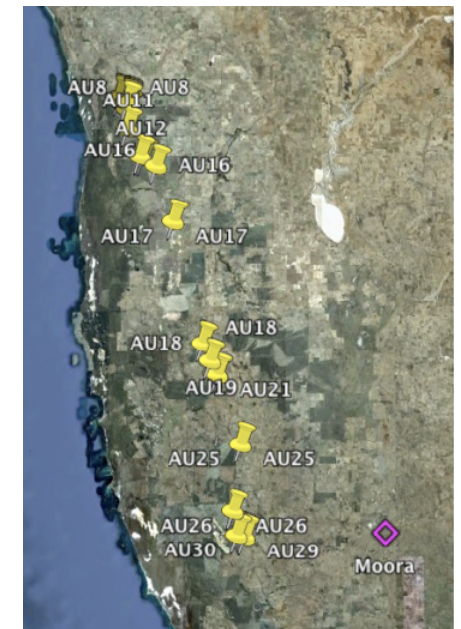

Supplement: S1 Fig — (PDF) [file pone.0130659.s001.pdf]

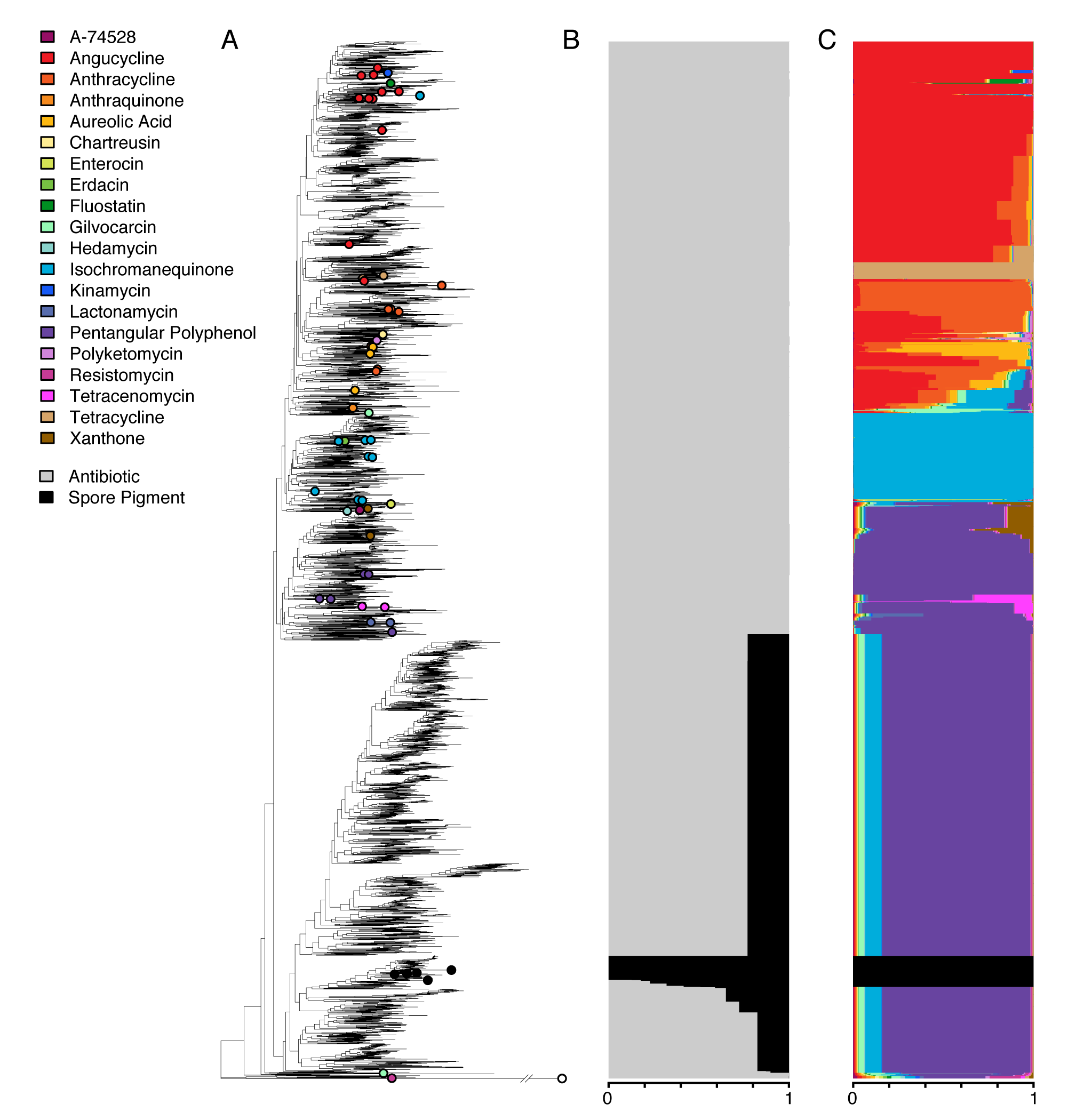

Supplement: S2 Fig — A) Phylogeny of all environmental KSα genes (black lines), along with antibiotic producing reference sequences (colored dots) and spore pigment producing reference sequences (black dots). B) Each bar indicates the inferred probabilities that the corresponding sequence codes for antibiotic production (in grey) or spore pigment production (in black). For example, a completely grey bar represents a sequence which probability to code for antibiotic production is 1. C) Each bar indicates the inferred probabilities that the corresponding sequence codes for each chemotype represented by the color (i.e. the length of a given colored band is proportional to the probability that the sequences codes for the chemotype represented by the color). Black bars indicate sequences which probability to encode spore pigmentation is greater than 0.5. Sequences from the top part of the tree encode diverse chemotypes. Most sequences from the bottom part of the tree likely encode yet-to-be discovered antibiotics rather than the inferred trait, given the lack of reference sequences in this part of the tree. (TIF) [file pone.0130659.s002.tif]

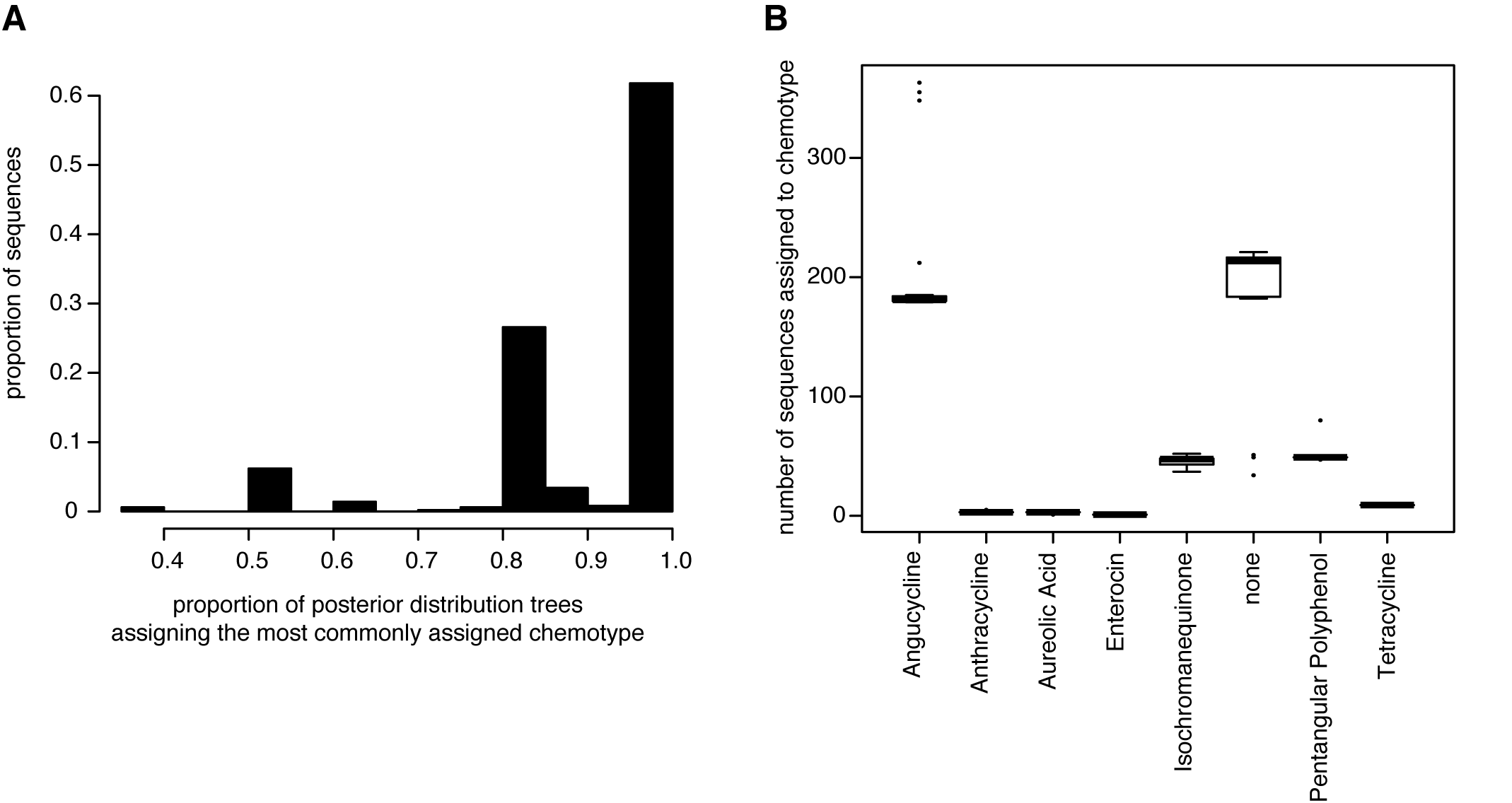

Supplement: S3 Fig — The trait assignment procedure is robust to phylogenetic uncertainty. A) Distribution across environmental sequences of the proportion of posterior distribution trees assigning the sequence to the most commonly assigned chemotype. For most sequences the most probable chemotype is consistent across phylogenies. B) Boxplot across posterior distribution trees of the number of sequences most probably coding for the given chemotype. “none” indicates that no chemotype was inferred with more than 0.5 confidence. The partitioning of sequences among chemotypes is consistent across phylogenies. (TIF) [file pone.0130659.s003.tif]

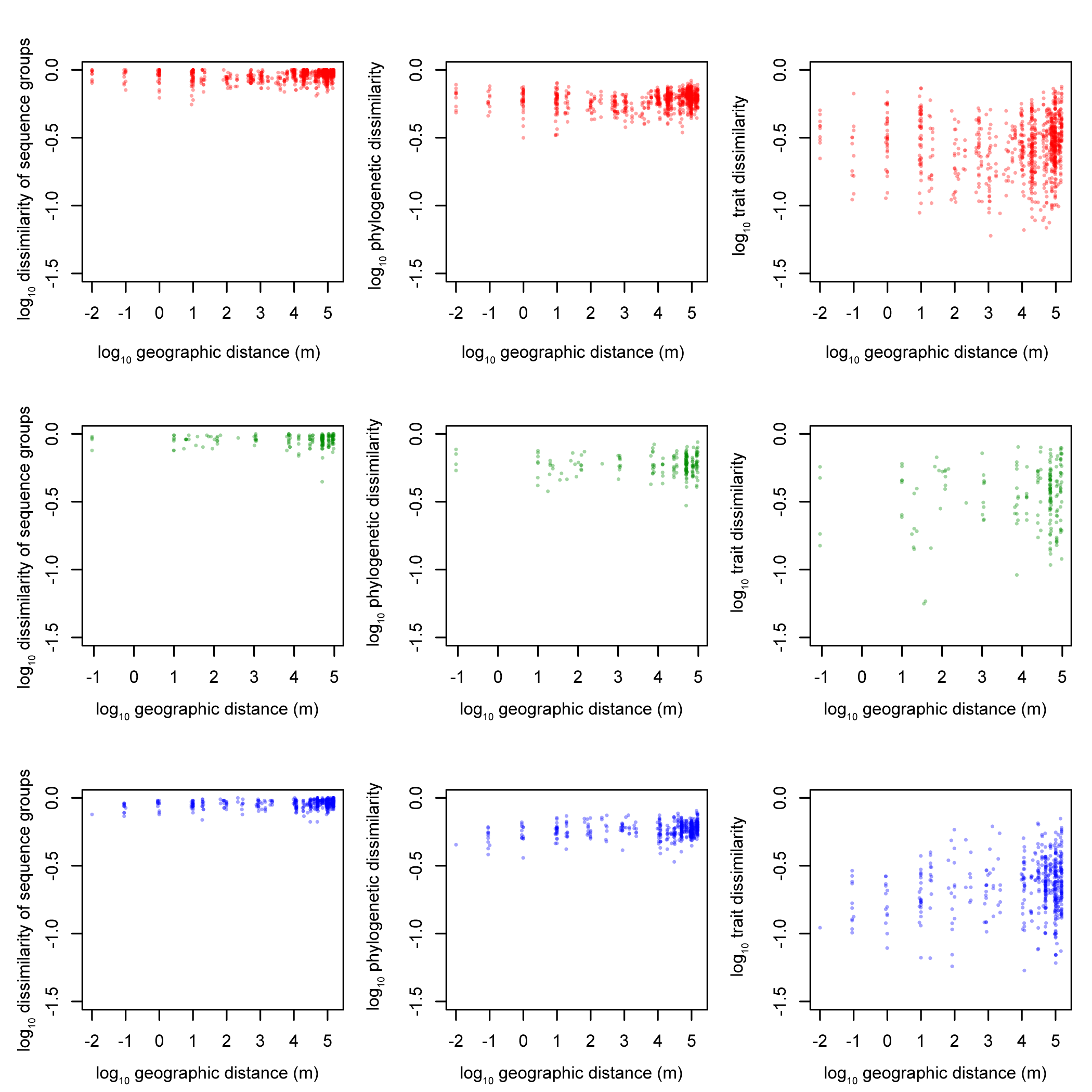

Supplement: S4 Fig — Comparisons of within-continents increase in community dissimilarity with geographic distance when diversity is measured in terms of sequence groups, phylogeny and traits. Analyses with the large uncharacterized clade excluded (see also S8 Fig) (TIF) [file pone.0130659.s004.tif]

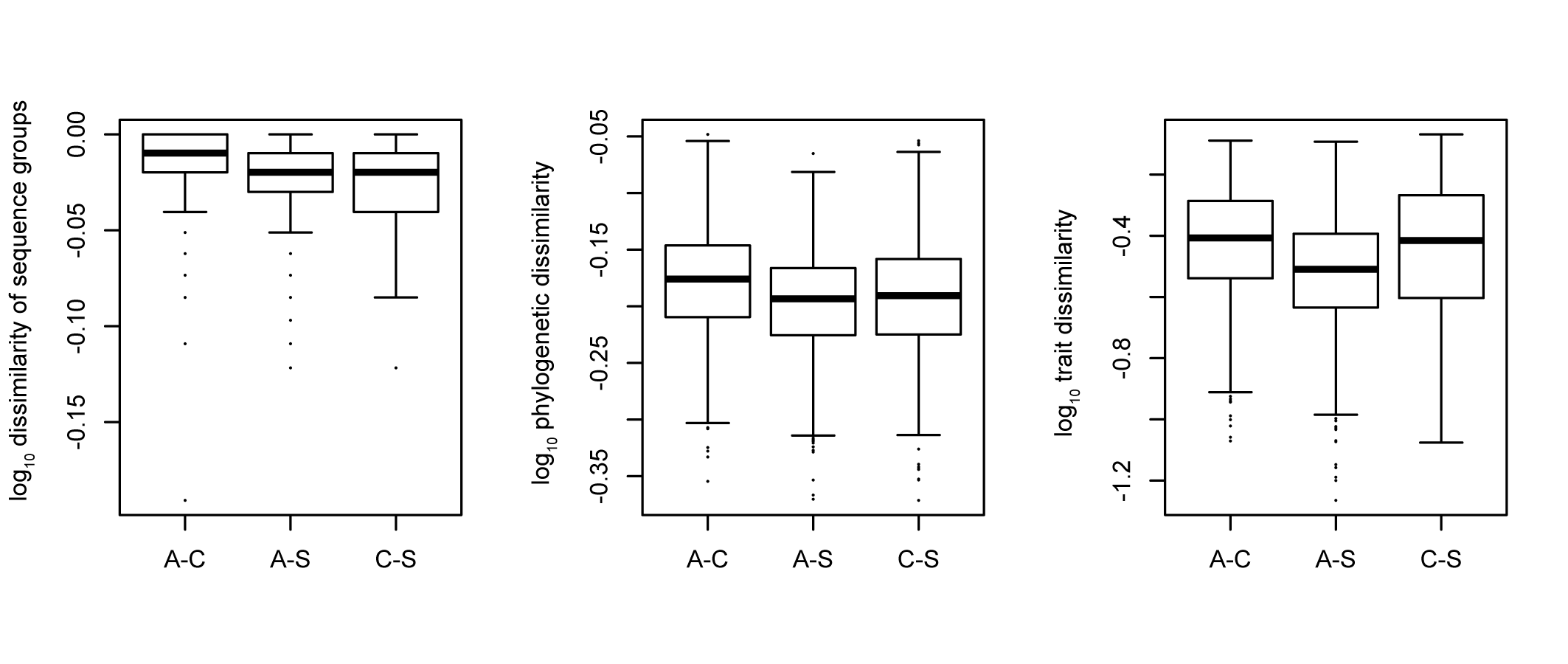

Supplement: S5 Fig — Boxplots of pairwise dissimilarity values, for measures of diversity based on sequence groups (top panel), phylogeny (middle panel) and traits (bottom panel). A-C: Australia / Chile; A-S: Australia / South Africa; C-S: Chile / South Africa. (TIF) [file pone.0130659.s005.tif]

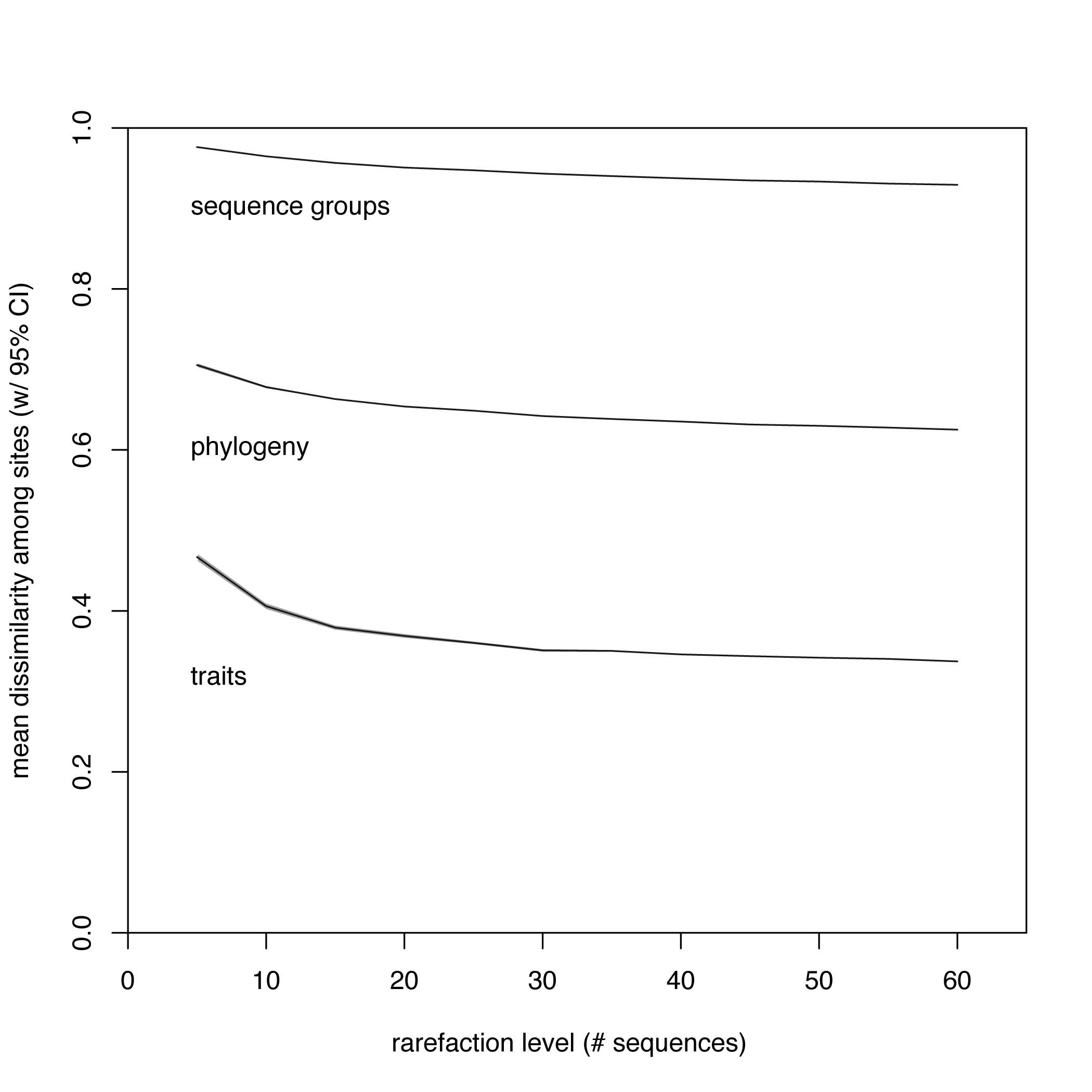

Supplement: S6 Fig — Plotted are mean (and 95% CI based on 50 replicate rarefactions) dissimilarity values (across all samples with at least 60 sequences) for taxonomic, phylogenetic and trait-based dimensions of biodiversity, as a function of the rarefaction level. CIs are very tight and can hardly be seen. Dissimilarity values asymptote very quickly with an increasing number of sequences. (TIF) [file pone.0130659.s006.tif]

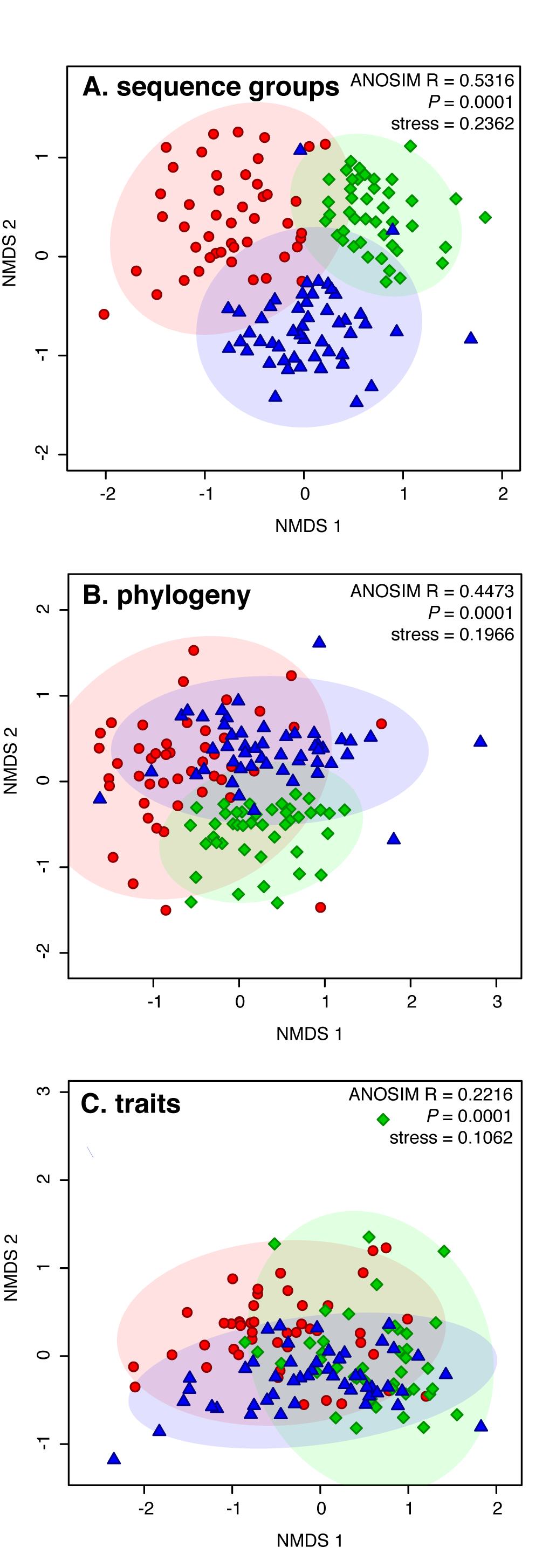

Supplement: S7 Fig — Results are consistent with results excluding sequences from the uncharacterized clade. (TIF) [file pone.0130659.s007.tif]

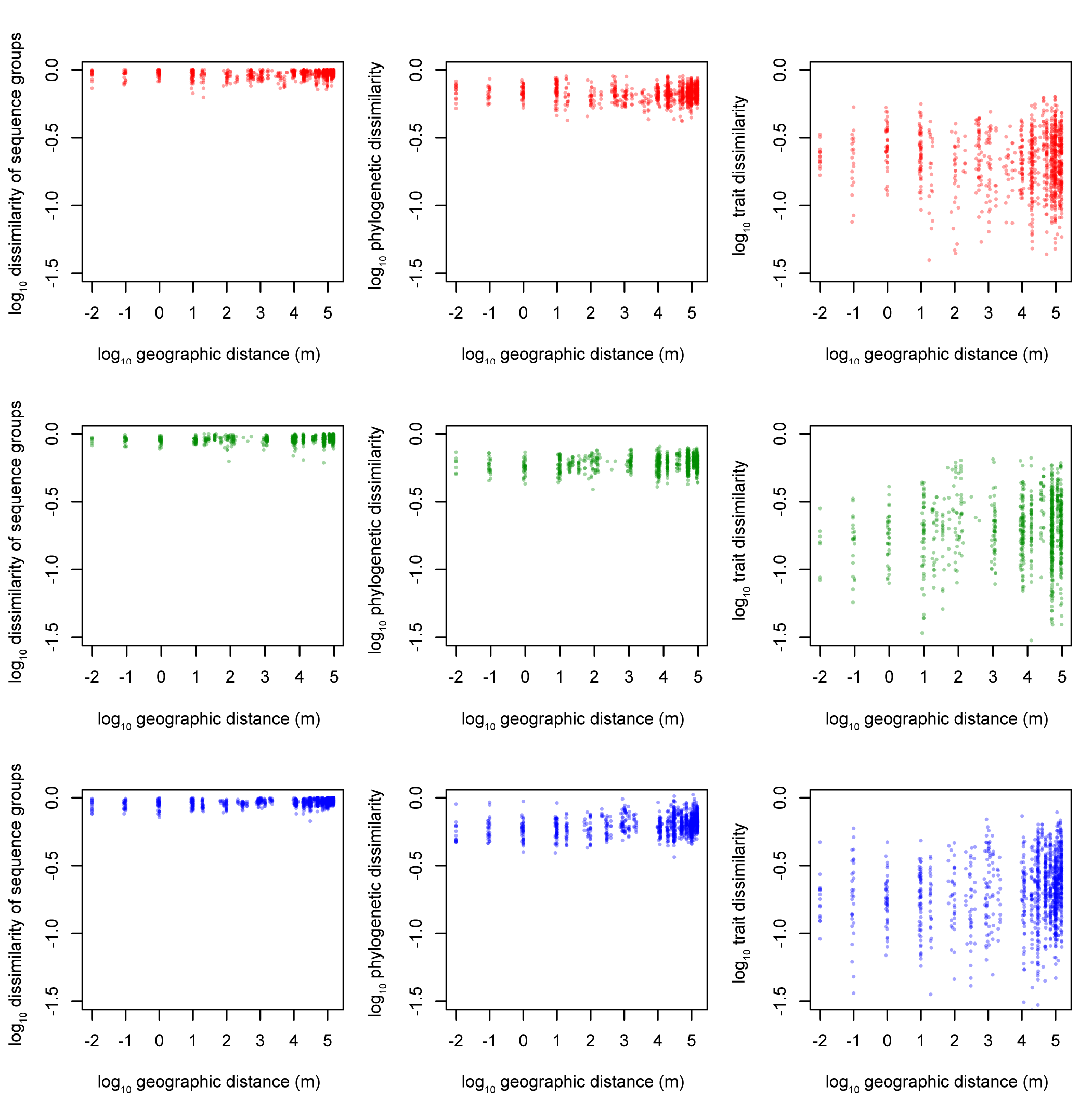

Supplement: S8 Fig — Results are consistent with results excluding sequences from the uncharacterized clade. (TIF) [file pone.0130659.s008.tif]
