# Supplementary material for: The Biogeography of Putative Microbial Antibiotic Production
Source: PLoS One. 2015 Jun 23;10(6):e0130659. doi: 10.1371/journal.pone.0130659 (PMC4478008; doi:10.1371/journal.pone.0130659)
Supplement: S1 Material & Methods — (DOC) [file pone.0130659.s009.doc]

**Supplementary Material & Methods**

*Data*

Soil cores were collected in the Mediterranean climate zone of Australia, Chile and South Africa (Fig. S1). On each continent, we collected 50 15-cm deep soil cores separated by geographic distances ranging from 1 cm to 170 km. Sampling sites were laid out along transects ranging between (30°42' S, 115°31' E) and (29°16' S, 115°06' E) in Australia, (34°22' S, 71°18' W) and (33°05' S, 71°09' W) in Chile, and (33°55' S, 19°11' E) and (32°27' S, 18°53' E) in South Africa (specific locations and more details are provided in (1)). We sampled in a relatively homogeneous flora and environment within each Mediterranean-type ecosystem. Specifically, cores were sampled on the same parent material and slope, aspect and fire history were kept as constant as possible. The elevation at which soil cores were collected was recorded in the field.

*DNA extraction*

We performed 5 replicate extractions of each soil sample, using a protocol amended from Zhou *et al.* (2). This protocol allowed us to extract DNA using the same method across the 3 distinct soil types found in each Mediterranean type system. For 6 out of the 150 samples (4 from Chile and 2 from Australia) we were unable to extract enough DNA to amplify KSα by PCR; these samples were eliminated from subsequent analyses.

For each replicate extraction, we weighed 0.2 g of soil into a bead beating tube from which we had removed and washed the buffer using sterile water so that only the beads remained (MoBio Labs). We added 480 µL of Buffer 1 (100 mM Tris-HCl, pH 8.0, 100 mM NaEDTA, pH 8.0, 100 mM NaH_2_PO_4_, 1.5 M NaCl and 1% CTAB), 2 µL of 20 mg mL^-1^ proteinase K (Applied Biosystems) and 7.2 µL of 175 mg mL^-1^ lysozyme (Sigma-Aldrich) to each tube and then performed a bead beating procedure using a MoBio vortex adapter at maximum vortex speed for 30 seconds. After bead beating, we placed the samples at 37^o^C on a platform shaker at 225 rpm for 30 mins. Following this incubation we performed a series of freeze-thaw extraction steps. We added 120 µL 20% SDS, 180 µL of 5M NaCl and 60 µL of 1.5M NaCl/5%CTAB to each tube and incubated the tubes for 10 min at 65^o^C. The sample was dipped in a dewar containing liquid N_2_, and then thawed again at 65^o^C for 15 min. Following one additional freeze-thaw step, we centrifuged the tubes at 13,000 rpm for 5 min and aseptically transferred the supernatant. To obtain the maximum amount of material, we then re-suspended the remaining pellet with 160 µL of Buffer 1 and 40 µL 20% SDS. We vortexed the pellets for 10 sec, incubated them at 65^o^C for 10 min and centrifuged at 13,000 rpm for 5 min. We removed the supernatant and combined it with the remainder of the sample supernatant.

Following this series of mechanical DNA extraction steps, we performed a series of phenol-chloroform and ethanol precipitation steps to chemically purify the DNA. We added 1 mL of phenol:chloroform:isoamylalcohol (25:24:1, Acros Organics) to the extracted supernatant, mixed and then centrifuged the tube for 5 min at 13,000 rpm. This created distinct aqueous and organic layers. We removed the aqueous layer and discarded the interface and organic layers. We repeated the phenol:chloroform:isoamylalcohol extraction steps two more times, removing the aqueous layer each time. Finally we added 700 µL of isopropanol and 300 µL of 10M C_2_H_7_NO_2_ (pH 7.5) and placed the samples at -80^o^C overnight. After cold incubation, we centrifuged the tubes for 30 min at 13,000 rpm, performed a 70% ethanol precipitation step and air dried the pellet. In the final step, we resuspended the pellet in 50 µL sterile DNAse-free water (VWR) and combined all replicates into 1 tube.

We ran 100 uL of DNA with 5 uL crystal violet through a 1% agarose gel in a gel tank that had been sterilized with 3% H_2_O_2_ to prevent contamination of the DNA. Two aliquots were run for each sample extracted. We ran the gels at 50V for 3 hours to ensure that DNA was fully separated from any contaminants. We stained the gel for 1 hour using crystal violet/methyl orange dyes and excised bands using sterile scalpel blades for each band. Finally, we extracted the DNA from agarose using a MoBio Gel Purification Kit using the manufacturers’ instructions for > 200 mg bands (MoBio Labs). We recombined the two aliquots following gel purification and stored the DNA at-80^o^C until PCR was performed.

*PCR*

Primer sequences were 5′-TSGCSTGCTTCGAYGCSATC-3′ forward and 5′-TGGAANCCGCCGAABCCGCT-3′ reverse (Integrated DNA Technologies, San Diego, CA, USA, S=C or G) and correspond to positions 6265 and 6877 of the type II PKS gene in *Streptomyces nogalater*, GenBank Accession Number Z48262 (3). Amplification with these primers thus results in a 612 bp product.

We used the following reaction conditions: 3 min at 94°C, 40 cycles at 94°C for 1 minute, 67.2°C for 1.5 min, 72°C for 1.5 min, followed by a 10 minute extension at 72°C. Given that several non-specific products were also amplified, we ran the full reaction volume on a 1% agarose gel containing SafeView stain (Gentaur, Brussels, Belgium) and excised the PKS-specific 612 bp-sized product from the gel. We pooled and cleaned the three replicate gel excisions together using a QiaQuick Gel Extraction Kit (Qiagen, Valencia, CA, USA).

*Cloning and sequencing*

We prepared clone libraries for each soil sample using the TOPO TA Cloning for Sequencing Kit (Invitrogen, Carlsbad, CA, USA) according to manufacturers’ instructions. At least 90 cloned amplicons per sample were sequenced from one direction at Functional BioSciences Inc. (Madison, WI, USA) using the Invitrogen T3 sequencing primer (Invitrogen, Carlsbad, CA, USA).

We used Sanger sequencing technologies, which, after trimming primers, resulted in 573 bp long sequences. More recent high-throughput technologies would have allowed much deeper sampling, but to the expense of sequence length and quality. Good quality long sequences result in more robust phylogenies; this was particularly important here, given the central role of the PKS phylogeny in our analyses.

All sequences were checked for putative chimeras using Bellorophon ((4) Huber-Hugenholtz correction, window size 200), and sequences identified as potential chimeras were removed from the analyses.

*Sequence alignment*

We manually checked trace data from each sequence and removed bad-quality or double calls in the sequences. This resulted in 573 bp-long sequences, with almost no variation in sequence length. A few of the sequences had 570-572 bp, most likely due to sequencing error; we aligned the sequences using Clustal (5) through the program BioEdit. The ~90 sequences from each of the 50 sampling locations were aligned individually, producing a 573 bp-long alignment. All of the individual alignments were then compiled into a single file, yielding a highly reliable alignment.

*Sensitivity of trait assignment to phylogenetic uncertainty*

To test the robustness of the trait assignment procedure to phylogenetic uncertainty, we performed sensitivity analyses using a Bayesian posterior distribution of trees from BEAST (6). Bayesian phylogenetic construction is too computer intensive to be performed on the full dataset; therefore, we performed sensitivity analyses for 500 sequences randomly sampled in the dataset. We used BEAST v. 1.7.5 with default parameters and log-normal uncorrelated relaxed clock to produce a posterior distribution of trees for these 500 sequences and the reference sequences. We then ran our trait assignment procedure on 100 trees randomly sampled from the posterior distribution of trees and checked, for each sequence, the robustness of trait assignment. Results are presented in Figure S3.

*Accounting for variation in PKS copy number*

We did not account for variation in PKS copy number in our study. As there is no *a priori* reason to believe that there is a correlation between copy number and the geographic distribution of organisms, the qualitative results should be robust to variation in copy number. A copy number correction method based on phylogenetic character inference has recently been proposed (7). We hope that this will encourage microbial ecologists to assemble gene copy number databases -- such as PKS copy number databases -- for reference organisms, using fully sequenced genomes (see (7) for an example using 16S). The utility of correcting for copy number variation in our trait centric (as opposed to taxa centric) study is debatable, as it could be argued that cells with several KS genes can potentially produce several different antibiotics and should be weighted more than cells with only one copy.

**References**

1. Morlon H et al. (2011) Spatial patterns of phylogenetic diversity. *Ecology Letters* 14:141–149.

2. Zhou J, Bruns MA, Tiedje JM (1996) DNA recovery from soils of diverse composition. *Appl Environ Microbiol* 62:316–322.

3. Metsä-Ketelä M et al. (1999) An efficient approach for screening minimal PKS genes from Streptomyces. *FEMS Microbiol Lett* 180:1–6.

4. Huber T, Faulkner G, Hugenholtz P (2004) Bellerophon: a program to detect chimeric sequences in multiple sequence alignments. *Bioinformatics* 20:2317–2319.

5. Larkin MA et al. (2007) Clustal W and Clustal X version 2.0. *Bioinformatics* 23:2947–2948.

6. Drummond AJ, Rambaut A (2007) BEAST: Bayesian evolutionary analysis by sampling trees. *BMC Evol Biol* 7:214.

7. Kembel SW, Wu M, Eisen JA, Green JL (2012) Incorporating 16S Gene Copy Number Information Improves Estimates of Microbial Diversity and Abundance. *PLoS Comput Biol* 8:e1002743.
