# Supplementary material for: The Biogeography of Putative Microbial Antibiotic Production
Source: PLoS One. 2015 Jun 23;10(6):e0130659. doi: 10.1371/journal.pone.0130659 (PMC4478008; doi:10.1371/journal.pone.0130659)
Supplement: S1 Table — (PDF) [file pone.0130659.s010.pdf]

accession #	polyketide	chemotype	chain length	first cyclization	priming unit	location
AB524586	hatomarubroigin	angucycline	C20	C7-C12	acetyl CoA	unknown
AF126429	jadomycin	angucycline	C20	C7-C12	acetyl CoA	Venezuela
AF080235	landomycin A	angucycline	C20	C7-C12	acetyl CoA	unknown
AJ632203	oviedomycin	angucycline	C20	C7-C12	acetyl CoA	unknown
AY228176S1	PD-116740	angucycline	C20	C7-C12	acetyl CoA	unknown
FJ670504	saquayamycin Z	angucycline	C20	C7-C12	acetyl CoA	Romania
AJ628018	sch 47554	angucycline	C20	C7-C12	acetyl CoA	Canada
AF324838	simocyclinone-D8	angucycline	C20	C7-C12	acetyl CoA	unknown
X87093	urdamycin	angucycline	C20	C7-C12	acetyl CoA	Tanzania
HQ828984	landomycin E	angucycline	C20	C7-C12	acetyl CoA	Arizona
GU134622	azicemicin A	angucycline	C20	C7-C12	aziridine carboxyl	unknown
DQ915964	aranciamycin	anthracycline	C20	C7-C12	acetyl CoA	unknown

AJ224512	nogalamycin	anthracycline	C20	C7-C12	acetyl CoA	unknown
AB024979	steffimycin	anthracycline	C20	C7-C12	acetyl CoA	unknown
AM156932	steffimycin	anthracycline	C20	C7-C12	acetyl CoA	unknown
AF257324	aclacinomycin	anthracycline	C21	C7-C12	propionyl CoA	Germany
AB024971	aclacinomycin	anthracycline	C21	C7-C12	propionyl CoA	Germany
AB008466	aklavinone	anthracycline	C21	C7-C12	propionyl CoA	Germany
AB024972	ciclacidine	anthracycline	C21	C7-C12	propionyl CoA	unknown
DQ280500	cosmomycin	anthracycline	C21	C7-C12	propionyl CoA	Brazil
STMDAUABCE	daunomycin	anthracycline	C21	C7-C12	propionyl CoA	unknown
STMDNRDPS	daunomycin	anthracycline	C21	C7-C12	propionyl CoA	unknown
AB024974	rhodomycin	anthracycline	C21	C7-C12	propionyl CoA	unknown
AF293442	R1128	anthraquinone	C18-C20	C7-C12	C3-C5 alkyl	unknown
AJ578458	chromomycin A3	aureolic acid	C20	C7-C12	acetyl CoA	Japan
X89899	mithramycin	aureolic acid	C20	C7-C12	acetyl CoA	unknown
HQ828986	UT-X26	aureolic acid	C20	C7-C12	acetyl CoA	Utah

FN565166	chrysomycin	gilvocarcin	C20	C7-C12	acetyl CoA	unknown
AY233211	gilvocarcin	gilvocarcin	C21	C7-C12	propionyl CoA	unknown
FN565485	ravidomycin	gilvocarcin	C21	C7-C12	propionyl CoA	unknown
SERDNABP	actinorhodin	isochromanequinone	C16	C7-C12	acetyl CoA	unknown
X63449	actinorhodin	isochromanequinone	C16	C7-C12	acetyl CoA	unknown
AL939122	actinorhodin	isochromanequinone	C16	C7-C12	acetyl CoA	unknown
X16144	granaticin	isochromanequinone	C16	C7-C12	acetyl CoA	California
X16300	granaticin	isochromanequinone	C16	C7-C12	acetyl CoA	California
AJ011500	granaticin	isochromanequinone	C16	C7-C12	acetyl CoA	California
GU233672	granaticin	isochromanequinone	C16	C7-C12	acetyl CoA	Vietnam
AB103463	medermycin	isochromanequinone	C16	C7-C12	acetyl CoA	unknown
AF098965	naphthocyclinone	isochromanequinone	C16	C7-C12	acetyl CoA	Illinois
EU852062	alnumycin	isochromanequinone	C18	C7-C12	butyryl CoA	Finland
AF058302	frenolicin	isochromanequinone	C18	C7-C12	butyryl CoA	unknown
STMFPS	frenolicin	isochromanequinone	C18	C7-C12	butyryl CoA	unknown
X77865	griseusin	isochromanequinone	C20	C7-C12	unknown	Peru

FJ719113	erdacin	erdacin	C16	C7-C12	acetyl CoA	Utah
AJ786382	chartreusin	chartreusin	C20	C7-C12	acetyl CoA	unknown
AY228174S2	kinamycin	kinamycin	C20	C7-C12	acetyl CoA	unknown
FJ483966	polyketomycin	polyketomycin	C20	C7-C12	acetyl CoA	unknown
AJ585192	resistomycin	resistomycin	C20	C9-C14	acetyl CoA	unknown
HM193369	fluostatin	fluostatin	C20	C7-C12	acetyl CoA	California
EU147299	lactonamycin	lactonamycin	C20	C9-C14	glycine	United Kingdom
EU147298	lactonamycin	lactonamycin	C20	C9-C14	glycine	Japan
AF254925	enterocin	enterocin	C21	C7-C12	benzoic acid	Hawaii
AY196994	hedamycin	hedamycin	C24	C7-C12	hexadienyl CoA	California
GU937384	A-74528	A-74528	C30	C9-C14	hexadienyl CoA	unknown
AF525490	fredericamycin	pentangular polyphenol	C30	C9-C14	hexadienyl CoA	unknown

D87924	pradimicin	pentangular polyphenol	C24	C9-C14	acetyl CoA	Fiji islands
AB019690	pradimicin	pentangular polyphenol	C24	C9-C14	acetyl CoA	Japan
EF151801	pradimicin	pentangular polyphenol	C24	C9-C14	acetyl CoA	Fiji islands
AF293355	rubroromycin	pentangular polyphenol	C26	C9-C14	acetyl CoA	unknown
AF509565	griseorhodin A	pentangular polyphenol	C26	C9-C14	acetyl CoA	unknown, marine
HQ828985	AZ154	pentangular polyphenol	C26	C9-C14	acetyl CoA	Arizona
AM501485	benastatin A	pentangular polyphenol	C28	C9-C14	hexanoyl CoA	unknown
AM900040	elloramycin	tetracenomycin	C20	C9-C14	acetyl CoA	unknown
STMTCREP	tetracenomycin C	tetracenomycin	C20	C9-C14	acetyl CoA	unknown

X15312	tetracenomycin C	tetracenomycin	C20	C9-C14	acetyl CoA	unknown
AJ630301	cervimycin	tetracycline	C19	C7-C12	malonamyl CoA	France
AB039379	chlorotetracycline	tetracycline	C19	C7-C12	malonamyl CoA	Missouri
Z25538	oxytetracycline	tetracycline	C19	C7-C12	malonamyl CoA	unkown
DQ143963	oxytetracycline	tetracycline	C19	C7-C12	malonamyl CoA	unkown
GQ409537	SF2575	tetracycline	C19	C7-C12	malonamyl CoA	Japan
AM492533	lysolipin	xanthone	C24	C9-C14	malonamyl CoA or acetyl	France
AB469194	FD-594	xanthone	C28	C9-C14	butyryl coA	unkown
AB024980	spore pigment	spore pigment	-	-	-	-
X62518	spore pigment	spore pigment	-	-	-	-

X55942	spore pigment	spore pigment	-	-	-	-
AB024981	spore pigment	spore pigment	-	-	-	-
AY485223	spore pigment	spore pigment	-	-	-	-
STMHYDROXY	spore pigment	spore pigment	-	-	-	-
AF293354	spore pigment	spore pigment	-	-	-	-
EG10274	outgroup	outgroup	-	-	-	-
